# Characterization of myelin compromise in rats with Alzheimer's‐like tauopathy

**DOI:** 10.1002/alz70855_100442

**Published:** 2025-12-23

**Authors:** Ai Liu

**Affiliations:** ^1^ McGill University, Montreal, QC, Canada

## Abstract

**Background:**

Alzheimer's disease (AD) affects both grey and white matter, leading to axonal degeneration, demyelination and glial pathologies. This study investigates the impact of pathological hyperphosphorylated tau (*p*‐Tau) on axonal damage and on demyelination and remyelination process in the Alzheimer's‐like tauopathy rat model in vivo.

**Method:**

We utilized the newly generated McGill‐R955‐hTau transgenic (Tg) rats expressing the longest human tau isoform with the P301S mutation. Cognitive function was assessed through behavioral testing. Tau expression patterns in grey and white matter were mapped using immunohistochemistry. Axonal damage and myelin pathology were examined via electron microscopy, and oligodendrocyte marker expression profiles were analyzed through immunohistochemistry.

**Result:**

Tg rats exhibited age‐dependent progression of *p*‐Tau levels, spreading from neuronal cell bodies in grey matter to axonal tracts and oligodendrocyte cytoplasm in white matter. This progression resulted in cognitive impairments, neurodegeneration, as well as axon and myelin loss. Ultrastructural analysis revealed extensive axonal and myelin degeneration characterized by swollen myelinated axons, empty myelin sheaths, splitting of myelin lamellae, formation of myelin balloons, and concentric membranous whorls. Myelin debris were observed in microglia and astrocytes. Following white matter demyelination, we found that oligodendrocyte progenitor cell (OPC) differentiated more efficiently in Tg rats than wild type (Wt) rats, however did not translate into increased remyelination due to increased axonal degeneration in Tg rats compared to Wt rats.

**Conclusion:**

Our findings suggest that axonal damage caused by abnormal tau accumulation and phagocytosis by microglia and astrocytes can lead to myelin pathology and loss. The resulting demyelination may signal OPCs to differentiate to facilitate remyelination.

